# Integrative radiogenomic analysis for multicentric radiophenotype in glioblastoma

**DOI:** 10.18632/oncotarget.7115

**Published:** 2016-02-01

**Authors:** Doo-Sik Kong, Jinkuk Kim, In-Hee Lee, Sung Tae Kim, Ho Jun Seol, Jung-Il Lee, Woong-Yang Park, Gyuha Ryu, Zichen Wang, Avi Ma'ayan, Do-Hyun Nam

**Affiliations:** ^1^ Department of Neurosurgery, Samsung Medical Center, Sungkyunkwan University School of Medicine, Seoul, Korea; ^2^ Samsung Biomedical Research Institute, Samsung Medical Center, Seoul, Korea; ^3^ Institute for Refractory Cancer Research, Samsung Medical Center, Seoul, Korea; ^4^ Samsung Advanced Institute of Technology, Samsung Electronics Co., Ltd., Seoul, Korea; ^5^ Department of Radiology, Samsung Medical Center, Sungkyunkwan University School of Medicine, Seoul, Korea; ^6^ Samsung Genome Institute, Samsung Medical Center, Seoul, Korea; ^7^ Department of Pharmacology and Systems Therapeutics, Icahn School of Medicine at Mount Sinai, New York, NY, USA

**Keywords:** glioblastoma, radiogenomic, multicentric

## Abstract

We postulated that multicentric glioblastoma (GBM) represents more invasiveness form than solitary GBM and has their own genomic characteristics. From May 2004 to June 2010 we retrospectively identified 51 treatment-naïve GBM patients with available clinical information from the Samsung Medical Center data registry. Multicentricity of the tumor was defined as the presence of multiple foci on the T1 contrast enhancement of MR images or having high signal for multiple lesions without contiguity of each other on the FLAIR image. Kaplan-Meier survival analysis demonstrated that multicentric GBM had worse prognosis than solitary GBM (median, 16.03 vs. 20.57 months, *p* < 0.05). Copy number variation (CNV) analysis revealed there was an increase in 11 regions, and a decrease in 17 regions, in the multicentric GBM. Gene expression profiling identified 738 genes to be increased and 623 genes to be decreased in the multicentric radiophenotype (*p* < 0.001). Integration of the CNV and expression datasets identified twelve representative genes: CPM, LANCL2, LAMP1, GAS6, DCUN1D2, CDK4, AGAP2, TSPAN33, PDLIM1, CLDN12, and GTPBP10 having high correlation across CNV, gene expression and patient outcome. Network and enrichment analyses showed that the multicentric tumor had elevated fibrotic signaling pathways compared with a more proliferative and mitogenic signal in the solitary tumors. Noninvasive radiological imaging together with integrative radiogenomic analysis can provide an important tool in helping to advance personalized therapy for the more clinically aggressive subset of GBM.

## INTRODUCTION

Glioblastoma (GBM) is the most common and lethal brain cancer. Recent advances in the molecular analysis of GBM have led to significant advance in our understanding of the molecular mechanisms of this disease [1]. However, the overall survival remains poor with a median survival of 15 months. GBM often spreads through an established route, such as commissural pathways, CSF channels, or through local extension by satellite formations. Multicentric GBMs have widely separated lesions that cannot be attributed to one of the aforementioned pathways. Thus, we postulated that multicentricity of the tumors represents a more invasive phenotype and has worse clinical outcome. Since Diehn, et al. first demonstrated the association between imaging features and genomic expression patterns in GBM [2], radiogenomic analysis has been recently introduced to identify imaging traits corresponding to different molecular phenotypes with clinical and biologic relevance [1–12]. Integrative analysis of multi-level molecular profiles for GBM and these imaging features can potentially provide new insights about the molecular mechanisms underlying the observed radiophenotype.

Thus, we postulated that multicentric GBMs have worse prognosis and have differential molecular signatures compared to other forms of GBM. To examine this hypothesis, we performed integrative analysis using RNA-seq analysis, and copy number variation (CNV) analysis and applied to patients with multicentricity phenotype of GBM.

## RESULTS

### Multicentric phenotype in GBM has worse clinical outcome than solitary GBM

A total of 20 out of the 51 patients with GBM were identified to have the multicentricity phenotype by MR imaging. We binary classified 20 patients as having multicentric GBM, and 31 patients having solitary GBM. Cox proportional hazard model and log-rank test demonstrated that multicentric GBM had worse prognosis than their counterparts (median, 16.03 vs. 20.57 months, *p* = 0.031) (Figure 2A). This assessment was based on the patients' MR contrast-enhancement of T1 weighted images and FLAIR images. We found that multicentric radiophenotype was closely associated with poor clinical outcome in the GBM patients. Other clinical parameters, including age, performance status, and extent of resection, were not significantly different between the groups.

**Figure 1:**
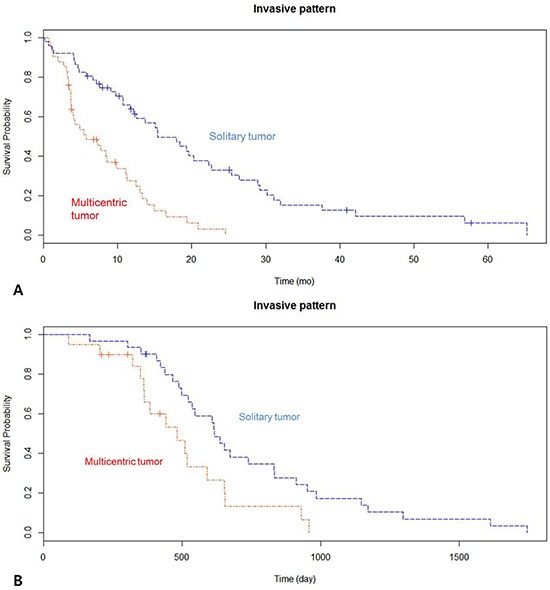
(**A**) Kaplan-Meier survival curves showing overall survival between the two groups in the 51 patients with newly diagnosed glioblastoma (GBM). (**B**) Kaplan-Meier survival curve demonstrating overall survival between the two groups in the TCGA dataset with newly diagnosed GBM.

**Figure 2:**
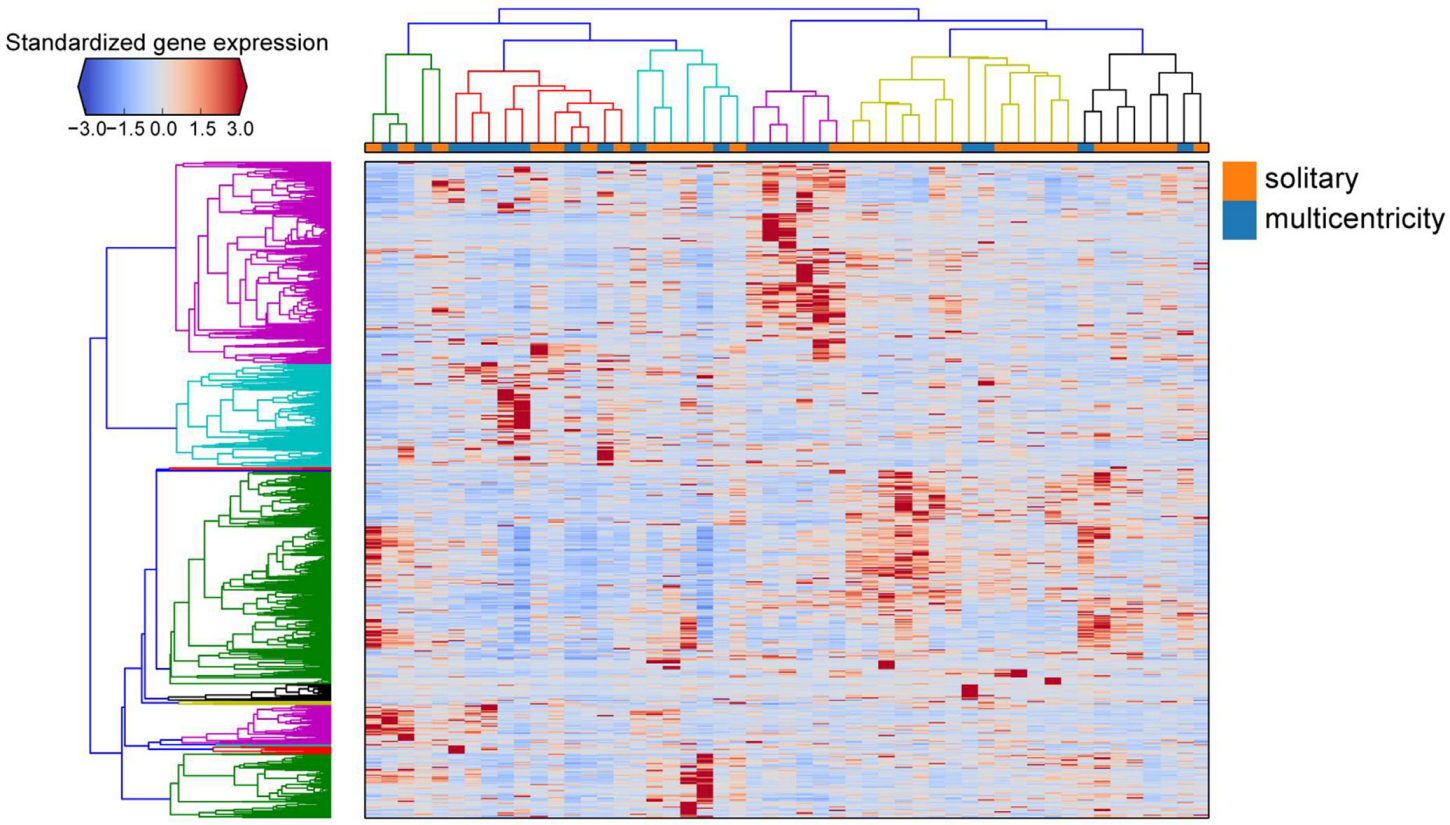
Hierarchical clustering of the gene expression matrix The standardized expression values of differentially expressed genes are shown in the heat map. Genes are clustered as the rows and patient samples are clustered as the column, with solitary and multicentricity tumor samples indicated by orange and blue, respectively.

### Identification of differentially expressed genes using RNA-seq

**Figure 3:**
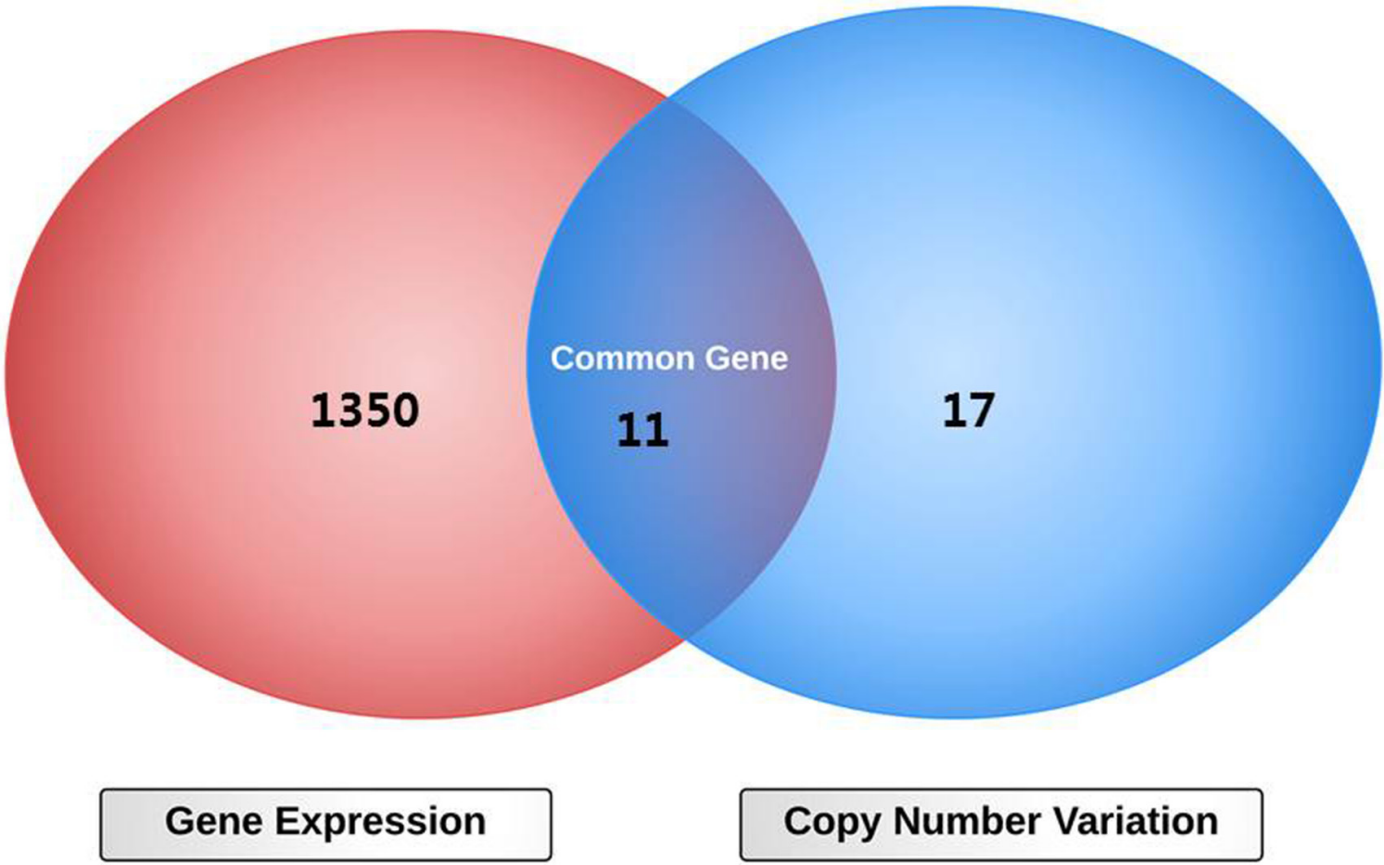
Diagram overlapping differentially expressed genes and altered copy number variation between multicentric and solitary radiophenotypes in glioblastoma

We sequenced 51 tumor tissue samples from GBM tumors, with each sample occupying one lane on an Illumina TrueSeq RNA Sample Prep kit. We applied DEGseq to identify the differentially expressed genes between both groups. We identified a signature of 1361 differentially expressed genes when comparing the multicentric radiophenotype with the solitary radiophenotype samples at an adjusted *p*-value(false discovery rate) < 0.001. Of these, 738 genes were increased and 623 genes were decreased in the multicentric radiophenotype compared to the solitary radiophenotype (Figure 3). Enrichment analysis of the decreased genes, comparing the multicentric GBM to the solitary GBM show lower expression of cell cycle genes and genes involved in glycolysis. Interesting enriched terms, for example, are genes involved in abnormal glia cells when knocked out in mice (MP0003436, *p*-value < 0.000005, Fisher Exact test), or direct interactors of the glucose transporter SLC2A4, *p*-value < 7.414e-23, Fisher Exact test. Full results can be accessed at: http://amp.pharm.mssm.edu/Enrichr/enrich?dataset=1ah9

On the other hand, genes that were decreasedin the multicentric GBM compared to the solitary GBM were enriched in extracellular matrix and cell adhesion components as well as immune response genes. Interesting enriched terms included high overlap with genes that cause abnormal nervous system (MP0003633, *p*-value < 4.1e-14, Fisher test) and the Lingula brain region from the Allen brain atlas (*p*-value < 2.365e-8, Fisher test). The Lingula wrinkle shape might use similar gene regulatory programs observed in multicentric GBM. Interestingly, the glucose transporter SLC2A4 was also enriched for direct interactors found in the increased genes. This suggests that regulation of glucose transport might be central to the difference between these two GBM subtypes. Complete analysis of the increased genes can be found at: http://amp.pharm.mssm.edu/Enrichr/enrich?dataset=1ahm

### Aberrant copy number variation between multicentric and solitary radiophenotypes in GBM

**Figure 4:**
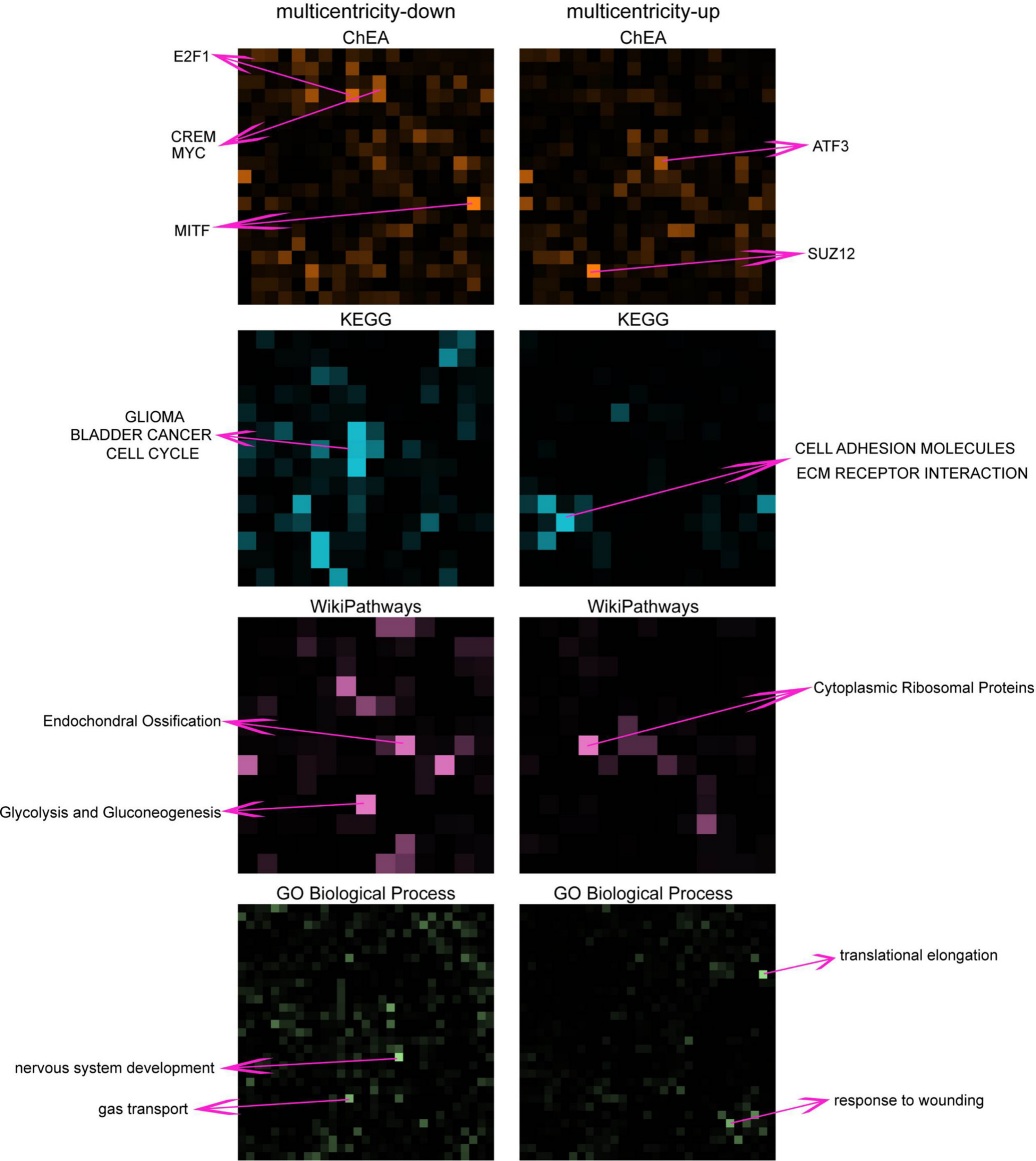
Visualization of enriched gene-sets in down- and up- regulated genes in multicentricity tumors over solitary tumors The enrichment of gene-sets for multicentricity and solitary tumors are shown in grids of different colors representing different gene-set libraries: ChEA, KEGG, WikiPathways and Gene Ontology Biological Process. Each square in the grid represents a gene-set and the brightness of the square positively correlate with the significance of the enrichment. Top enriched gene-sets are annotated.

CNV analysis was performed using Agilent SurePrint G3 Human CGH 4 × 180 k arrays. Each tumor tissue was compared to normal tissue from the same individual. 11 and 17 chromosomal regions were found to be significantly increased and decreased in copy number in the multicentric radiophenotype compared to the solitary radiophenotype, respectively. Structural genetic variation, such as CNAs, can critically is correlated with gene expression and contributes to significant phenotypic variation [23, 24]. To identify the significant genes that exhibited concordant CNV and gene expression changes, we overlapped the two datasets as presented in the Venn diagram (Figure 4). Decreased copy number was more prevalent at 7q31.1, 12q14.3, and 13q34 in the multicentric radiophenotype. Gain was more prevalent at 7q23.1, 7q21.1, 10q23.1, and 12q14.1 in the multicentric phenotype. Eleven representative genes: CPM, LANCL2, LAMP1, GAS6, DCUN1D2, CDK4, AGAP2, TSPAN33, PDLIM1, CLDN12, and GTPBP10 demonstrated high correlations between copy number variation and gene expression in the tumor tissues.

### Differential gene expression profiles reveal activation of the extracellular matrix receptor interaction pathway in GBM with invasive radiophenotype

**Figure 5:**
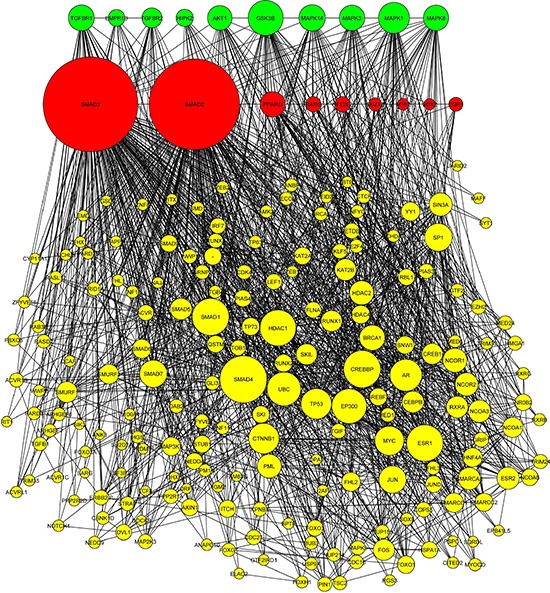

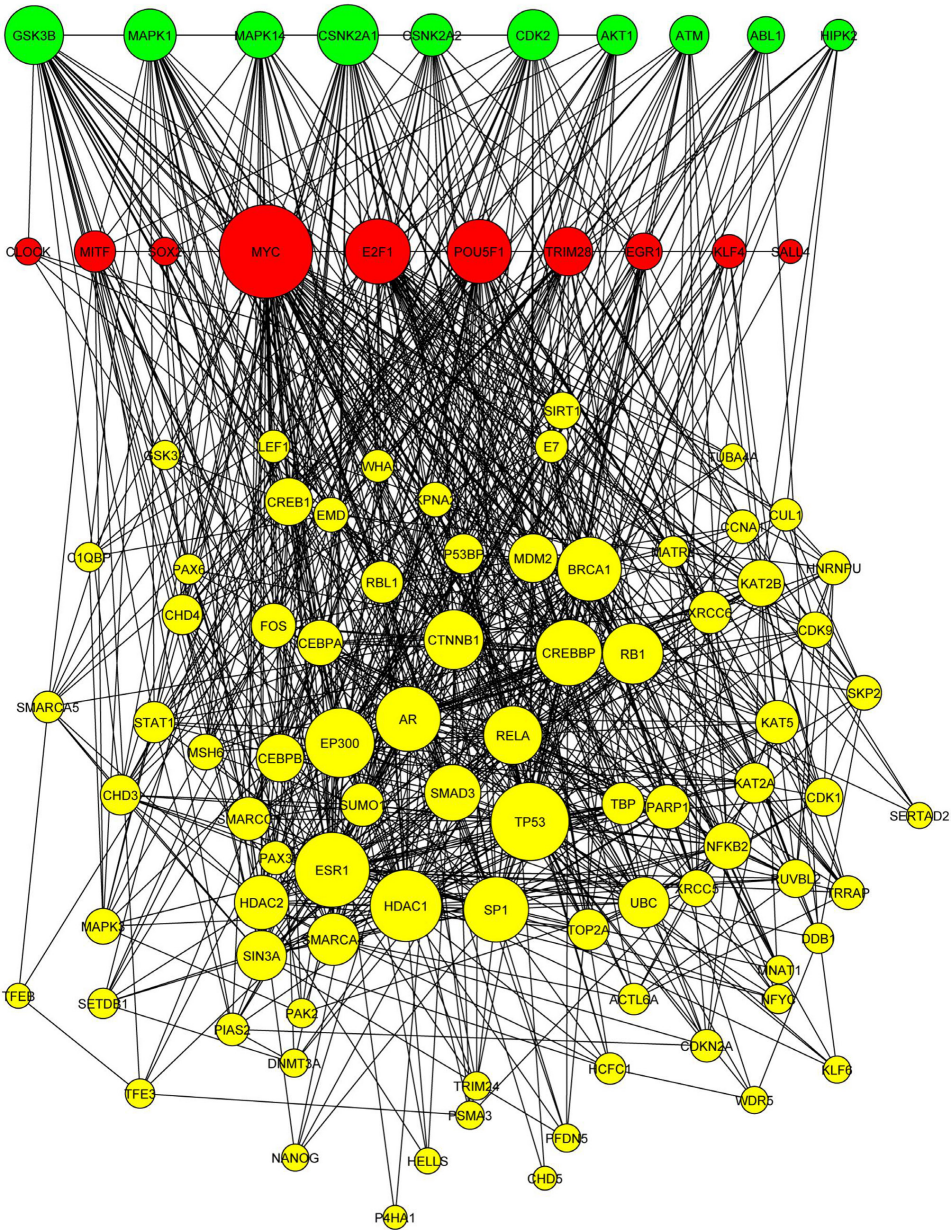
Network analysis using Expression2Kinase (**A**) The transcription factors (red nodes), kinases (green nodes), and intermediate proteins (yellow nodes) predicted as top candidates to regulate the expression of the up-regulated genes in multicentric GBM compared with solitary. (**B**) The transcription factors, kinases, and intermediate proteins predicted for down-regulating genes with the more invasive phenotype.

**Figure 6:**
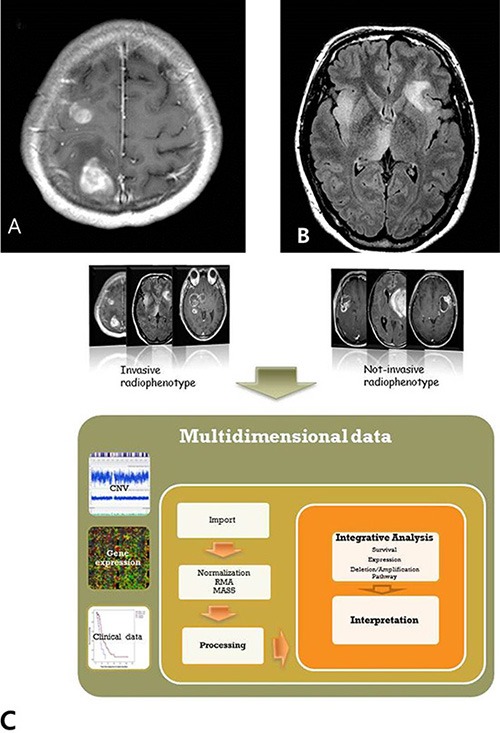
Multicentric phenotypes defined in glioblastoma (**A**) Multiple enhancing tumors on T1 contrast enhancement MR image. (**B**) Multiple infiltrative lesions without continuity on the FLAIR image. (**C**) Schematic diagram of this study.

To obtain a more global mechanistic view of the altered biological pathways that could be responsible for invasiveness in GBM, we performed network analysis using the Expression2kinases (X2K) software [21]. In the first step of the X2K analysis, the lists of up- and down-regulated genes were used as input for enrichment analysis to generate a list of predicted upstream transcription factors. The transcription factor: SOX2, SUZ12, SMAD3, SMAD2, EGR1, PPARG, RARG, MTF2, AND NFE2L2 were predicted as top candidates that potentially regulate the expression of the up-regulated genes; and the transcription factors: E2F1, MYC, KLF4, POU5F1, SOX2, MITF, CLOCK, SALL4, EGR1 and TRIM28 were predicted as regulators of the down-regulation genes in the invasive phenotype (Figure 5). While there is some overlap among these transcription factors, the predicted kinases upstream of these factors point clearly to two distinct processes. Nuclear kinases and casein kinases are enriched for the down regulated genes, consistent with the enrichment for cell cycle genes, whereas TGF beta receptors and other MAPK kinases are enriched for the upregulated genes. GSK3B, MAPK1 and HIPK2 are shared among both up and down predicted pathways. Overall these networks provide additional view of the potential regulatory mechanisms that differentiate the multicentric from the solitary GBM and potentially point to future drug targets (Figure 6).

### Validation of differentially expressed genes signatures in independent cohorts

To validate the differentially expressed genes signature in independent cohorts of patient samples, we used publicly available microarray data from the TCGA dataset. 166 of a total 508 GBM samples had corresponding imaging data from The Cancer Imaging Archive. From the 166 patients with primary GBM who had available clinical, genomic and radiological data, we binary classified 39 patients as having multicentric GBM, and 127 patients having solitary GBM using preoperative MR imaging. Cox proportional hazard model and log rank test revealed that GBM patients with multicentric radiological phenotype had worse prognosis than those with solitary phenotype (median 7.23 vs. 15.06 months, *p* < 0.0001) (Figure 2B). Based upon a predefined set of molecular markers specific to the multicentric group, we predicted the TCGA microarray dataset into two groups using the nearest template prediction (NTP) method with statistical significance (false discovery rate, FDR < 0.2). As a result, we found 76.9% (30 of 39) samples in the multicentric group represented predefined markers of multicentric group, while 60.6% (77 of 127 patients) in the solitary group showed them (Pearson Chi-square test, *p* = 0.063).

## DISCUSSION

The dismal prognosis of GBM is attributed to the invasiveness of GBM cells to infiltrate surrounding brain parenchyma. Such invasiveness is the major cause of tumor recurrence or progression. The tumors with the most aggressive invasive phenotype are more likely to have multiple tumor foci (multicentricity) compared with the other forms of GBM.

Multicentric gliomas are well-separated lesions, localized in different lobes or hemispheres, without anatomical continuity between lesions [25–27]. Overall incidence of multifocal or multicentric glioblastoma ranges from 16.2 to 35% at the time of initial diagnosis, which is higher than was previously considered [25–28]. Continuous advances in MR imaging technologies have contributed to the increase diagnosis of multiplicity in gliomas. A growing number of studies supported that changes in T2-weighted images or FLAIR sequences can reflect modifications in the extracellular matrix by invading glioma cells [28–30]. A recent study by Patil, et al. [31] suggested that multicentricity GBM was biologically different from single lesion disease and spreads more quickly, leading to worse survival. Therefore, we hypothesized that multiple lesions in GBM can represent more invasive phenotype and have their own underlying molecular genomic characteristics.

To account for this complex relationship between multiple genomic profiles and invasive phenotype in GBM, we performed integrative analysis of multicentric radiophenotype imaging and genomic data including gene expression and CNV profiling. First, we found that multicentric radiophenotype in GBM was closely associated with overall survival as observed in two independent GBM cohorts (TCGA and Samsung Medical Center). This finding was consistent with the concept that multicentric GBM represents a more aggressive and invasive phenotype, leading to poor clinical outcome. We then determined the relationship between invasive imaging phenotype and the respective gene-expression profiles assessed by next generation RNA-seq analysis.

High-throughput sequencing technology is rapidly becoming the standard method for measuring RNA expression levels [32, 33]. Finding genes that are differentially expressed between the two radiophenotypes is an integral step toward understanding the molecular basis of this phenotypic variation. Combined with copy number alterations detected by CGH arrays we provided a comprehensive view to discover underlying candidate genes. As CNV harboring duplications and deletions potentially lead to changes in gene expression levels [34–36]. Concordance between RNA gene expression levels and CNV gene dosage has been found in several genes in multiple cancers [37–41]. Accordingly, integrated analysis of radio-genomic data can discover copy number alterations and their possible regulatory effects on gene expression in the specific imaging phenotype [42–44].

In this study, CGH arrays revealed that chromosomes at 7q, 12q and 13q had decreased copy number compared with non-invasive phenotype. Gene expression profiles demonstrated that up-regulated genes in the multicentric phenotype were associated with cell adhesion and cell-to-cell interactions functions. Among eleven representative genes showing high correlations between copy number and gene expression in this study, LAMP1 is a late endosomal/lysosomal marker [45, 46] associated with tumor cell motility and invasiveness. GAS6, major ligand of AXL receptor tyrosine kinase, is also notable because it has been demonstrated to be overexpressed and activated in many human cancers such as lung, breast, and pancreatic, and have been correlated with poor prognosis, promotion of increased invasiveness and metastasis, the EMT phenotype and drug resistance [47–51]. AGAP2 belongs to the centaurin gamma-like family. It mediates anti-apoptotic effects of nerve growth factor by activating nuclear phosphoinositide 3-kinase. AGAP2 is overexpressed in cancer cells, and promotes cancer cell invasion [41, 52, 53]. In the future, those characteristic molecular candidates can be further investigated to be closely involved with the tumor invasiveness and as such are great candidates for future *in-vitro* and *in-vivo* studies. In conclusion, integrative radiogenomics analysis provides more in-depth knowledge about the genomic landscape of glioblastoma.

## MATERIALS AND METHODS

### Patient population

Between May 2004 and June 2010, 51 GBM tumor samples with available clinical and pathology reports were obtained from the Samsung Medical Center data registry (SMC, Seoul, Korea). Recurrent tumors, secondary GBM, previous history of treatment and those without comprehensive clinical information were excluded from this study. All tissue samples were previously untreated surgical specimens from patients with grade IV gliomas, which were histologically confirmed grade IV GBM according to the World Health Organization (WHO) classification [13] and collected with written informed consent under a protocol approved by the Institutional Review Board of the Samsung Medical Center (2010-04-004, Seoul, Korea). Median age of the patients was 53 years (range, 29-74 years) and patients were composed of 31 males and 20 females. The metadata about the 51 GBMs samples are provided in Table S1.

To validate the prognostic value of our multicentric radiophenotype in the primary dataset, we used the original material and data provided by The Cancer Genome Atlas (TCGA) [14] and corresponding imaging data from The Cancer Imaging Archive (TCIA) [15, 16], which are publicly available resources containing multidimensional genomic and clinical information about GBM before 2014. We first downloaded the DICOM files from TCIA GBM database and analyzed their MR data with the open-source OsiriX software (http://www.osirix-viewer.com/). All MR images were acquired by using the same imaging protocol as described above. Using clinical data from the TCGA dataset based upon this classification of multicentricity, we performed survival analysis on 230 patients with primary GBM (treatment naive GBM).

### MR imaging protocol

MR imaging in this study was conducted on a 1.5 T and 3.0 T scanner and included T1-weighted, T2-weighted, and fast-spin echo sequences. Post-contrast images were acquired 5 minutes after contrast agent injection. The standard MRI protocol included axial T1-weighted imaging, T2-weighted imaging, and fluid-attenuated inversion recovery (FLAIR), perfusion-weighted MR images.

### Definition of multicentric GBMs on MR finding

All MRI exams were performed on a 1.5 T Sigma Echospeed scanner (GE Medical Systems). All GBMs were binary classified as multicentric or solitary phenotype by their radiological MR characteristics. Multicentricity of the tumor on MR images was defined as the presence of multiple foci on the T1 contrast enhancement image (Figure 1A), or high signal multiple lesions without contiguity of each other on the FLAIR image (Figure 1B). All images were evaluated by consensus in a blinded fashion by two board-certified radiologists (ST Kim, 24 year-experience & JH Cha 4 year-experience). Both readers were blinded to the genomic signatures and other clinical details at the time of image interpretation.

### Survival analysis

Overall survival (OS) was defined as the time between the date of pathological diagnosis and the date of death or the date of last clinical follow-up. The univariable Cox proportional hazards model was used to determine hazard ratios (HRs) of each variable as a predictor of OS. Kaplan-Meier survival analysis was performed using R 3.0.1 (Vienna, Austria; http://www.R-project.org/) and *p*-value < 0.05 was deemed statistically significant.

### Comparative genome hybridization array (CGH)

For 32 of a total of 51 patients, CGH array data was collected. DNA was extracted using the DNeasy kit. CGH arrays were applied using Agilent SurePrint G3 Human CGH 4 × 180 k arrays, according to the manufacturer's instructions. CGH FE files were processed and normalized, using the Agilent Genomic WorkBench 7.0.4.0. The DNAcopy R package was used to estimate DNA copy number for genomic segments. From the segmentation data, the copy number of each gene was calculated, by averaging copy numbers of all exonic segments of each gene.

### Next-generation RNA-sequencing (RNA-Seq)

RNA-Seq was performed for all the 51 patients. RNA-Seq based transcriptome profiling was performed by the Samsung Institute for Intractable Cancer Research (Seoul, Korea), using the Illumina TrueSeq RNA Sample Prep kit. For the samples subjected to RNA-seq, we isolated 5 μg of total RNA from each sample. For quantitation of mRNA abundance, sequenced reads in FASTQ files were trimmed to include only 30 nucleotides from the 5′ end of each read. The trimmed reads were aligned on the human reference genome (hg19) using GSNAP, not allowing any mismatches, indels, or splicing variants. The resulting alignment SAM files were sorted and summarized into BED files using SAMtools and bedTools (bamToBed). The DEGseq R package was used to calculate RPKM (Reads Per Kilobase per Million mapped reads) from the hg19 refFlat file downloaded from the UCSC genome browser and the BED files that were generated during the per nucleotide coverage analysis. Log transformation was applied to correct for the skewed distribution.

To identify the differentially expressed genes in the multicentric GBMs, DEGseq was used. The input to DEGseq was uniquely mapped reads from the RNA-seq data with a gene annotation of the corresponding gene expression values. The DEGseq R package MA-plot-based method was used to estimate the noise level by comparing technical replicates in the data by integrating the Fisher's exact test and likelihood ratio tests [17, 18]. *P*-values calculated for each gene are adjusted to *Q*-values for multiple hypotheses testing with Benjamini and Hochberg (BH) [19] or Storey [20] correction methods.

### Integration of copy number variation and genome-wide expression analyses

Data from copy number variation (CNV) and genome-wide expression profiles were analyzed individually (Figure 1C). To identify the significant genes that exhibited CNV and gene expression changes, we overlapped the two datasets as presented in the Venn diagram. Thus, correlation between mRNA and CNV signatures was performed for all 32 patients having CNV and expression data. Subsequent analysis and correlation with the imaging features was performed for all patients as well.

### Pathway analysis

The dataset containing differentially expressed genes was uploaded into the Expression2Kinases (X2K) software [21]. The first step of the X2K computational pipeline is to perform gene-set enrichment analysis, using the ChIP-seq/chip Enrichment Analysis (ChEA) database on the differentially expressed genes to identify the likely transcription factors that are responsible for the observed changes in expression. The Fisher exact test (*p* < 0.05, with BH correction) was used to assess the significance of the associations between the list of differentially expressed genes and targets of the transcription factors based on prior ChIP-Seq published studies. The next step in the X2K pipeline is to connect the enriched transcription factors using known protein-protein interactions. Using the shortest path algorithm and a collection of online protein-protein databases, a subnetwork is constructed to expand the set of transcription factors to its local protein interaction neighborhood. Finally, the X2K pipeline applies kinase enrichment analysis applied to all the proteins in the subnetworks, to predict the most likely upstream protein kinases that are involved in the observed changes in gene expression. The kinase enrichment analysis is performed using the kinase enrichment analysis (KEA) module within X2K with the same statistical test as the enrichment analysis for the transcription factors.

### Other statistical analyses

Hierarchical clustering of the gene expression matrix was performed by taking the RPKMs of the 1,361 differentially expressed genes identified by DEGseq [1] across all 51 patient tumor samples. Correlation distance was used to measure the dissimilarities between genes and samples. The average linkage function was used for agglomerative clustering. Enrichment analysis of the lists of differentially expressed genes was performed using Enrichr [2] to examine the overlap of genes across multiple gene-set libraries. The enriched gene-sets were visualized using Rubik (http://amp.pharm.mssm.edu/rubik/). Most other calculations were performed using the R language and the R statistical programming environment with Bioconductor packages. GC content was calculated using the DEGseq package [22].

## SUPPLEMENTARY MATERIAL TABLE




