# Supplementary material for: Integrative radiogenomic analysis for multicentric radiophenotype in glioblastoma
Source: Oncotarget. 2016 Feb 1;7(10):11526–38. doi: 10.18632/oncotarget.7115 (PMC4905491; doi:10.18632/oncotarget.7115)
Supplement: Supplementary file 1 [file oncotarget-07-11526-s001.pdf]

Integrative radiogenomic analysis for multicentric radiophenotype in glioblastoma

Supplementary Materials

Supplementary Table 1: Clinico-pathological characteristics in patients with glioblastoma
