# Supplementary material for: Integrative radiogenomic analysis for multicentric radiophenotype in glioblastoma
Source: Oncotarget. 2016 Feb 1;7(10):11526–38. doi: 10.18632/oncotarget.7115 (PMC4905491; doi:10.18632/oncotarget.7115)
Supplement: Supplementary file 2 [file oncotarget-07-11526-s002.doc]

Supplementary Table S1: Clinico-pathological characteristics in patients with glioblastoma

| No. | sample ID | Sex | Age | Surgery date | pathology | RNAseq | aCGH | Location (described) | Multicentricity (multicentric 1, solitary 0) | MGMT status  (unmethylated 0, methylated 1, unidentified 2) | IDH-1 (wild 0, mutated 1, unidentified 2) | Extent of resection | Molecular  subtype (classical 1, proneural 2, neural 3, mesenchymal 4) | CCRT | Survival status (death 1, alive 0) | OS |
| --- | --- | --- | --- | --- | --- | --- | --- | --- | --- | --- | --- | --- | --- | --- | --- | --- |
| 1 | IRCR-GBM-001 | F | 63 | 2013-12-05 | Glioblastoma with oligodendroglioma component | O | O | Rt F | 1 | 1 | 0 | PTR | 3 | 1 | 1 | 414 |
| 2 | IRCR-GBM-002 | F | 51 | 2010-09-01 | Glioblastoma | O | O | Rt F | 1 | 1 | 0 | PTR | 3 | 1 | 1 | 481 |
| 3 | IRCR-GBM-003 | M | 46 | 2010-09-29 | Glioblastoma | O | O | Rt P | 1 | 1 | 0 | PTR | 3 | 0 | 1 | 202 |
| 4 | IRCR-GBM-004 | M | 53 | 2011-04-27 | Glioblastoma with oligodendroglial component | O | O | Rt T | 1 | 1 | 0 | PTR | 4 | 1 | 1 | 655 |
| 5 | IRCR-GBM-005 | M | 45 | 2011-06-14 | Glioblastoma | O | O | Lt F | 1 | 0 | 0 | GTR | 3 | 1 | 1 | 350 |
| 6 | IRCR-GBM-006 | F | 52 | 2011-08-30 | Glioblastoma | O |  | Lt. P | 1 | 0 | 0 | GTR | 4 | 0 | 1 | 442 |
| 7 | IRCR-GBM-007 | F | 56 | 2011-09-14 | Glioblastoma with oligodendroglioma component | O |  | Lt T | 1 | 1 | 0 | PTR | 2 | 1 | 1 | 511 |
| 8 | IRCR-GBM-008 | F | 54 | 2012-10-18 | Glioblastoma | O |  | Rt P | 1 | 2 | 0 | GTR | 2 | 1 | 1 | 653 |
| 9 | IRCR-GBM-009 | M | 46 | 2013-10-31 | Glioblastoma | O |  | Rt F | 1 | 2 | 0 | GTR | 2 | 1 | 0 | 419 |
| 10 | IRCR-GBM-010 | M | 43 | 2013-09-25 | Glioblastoma | O |  | Rt P | 1 | 1 | 0 | PTR | 1 | 1 | 0 | 304 |
| 11 | IRCR-GBM-011 | F | 60 | 2013-12-30 | Glioblastoma with oligodendroglioma component | O |  | Lt T | 1 | 1 | 0 | PTR | 1 | 1 | 0 | 209 |
| 12 | IRCR-GBM-012 | F | 40 | 2007-02-14 | Glioblastoma with oligodendroglioma component | O | O | Rt F | 1 | 2 | 0 | GTR | 1 | 0 | 1 | 363 |
| 13 | IRCR-GBM-013 | M | 41 | 2007-03-22 | Glioblastoma | O | O | Rt F | 1 | 2 | 0 | PTR | 3 | 1 | 1 | 959 |
| 14 | IRCR-GBM-014 | F | 55 | 2007-03-28 | Glioblastoma | O |  | Lt P | 1 | 2 | 0 | PTR | 2 | 0 | 1 | 518 |
| 15 | IRCR-GBM-015 | M | 29 | 2007-06-12 | Glioblastoma | O | O | Lt F | 1 | 2 | 0 | GTR | 1 | 1 | 1 | 930 |
| 16 | IRCR-GBM-016 | M | 48 | 2007-07-30 | Glioblastoma | O | O | Lt T | 1 | 2 | 0 | PTR | 1 | 0 | 1 | 90 |
| 17 | IRCR-GBM-017 | F | 61 | 2008-07-31 | Glioblastoma | O | O | Rt F | 1 | 2 | 0 | GTR | 4 | 0 | 1 | 591 |
| 18 | IRCR-GBM-018 | M | 43 | 2008-09-15 | Glioblastoma | O | O | Lt T | 1 | 2 | 0 | GTR | 4 | 1 | 1 | 383 |
| 19 | IRCR-GBM-019 | F | 45 | 2009-03-19 | Glioblastoma | O | O | Rt T | 1 | 2 | 0 | GTR | 4 | 1 | 1 | 322 |
| 20 | IRCR-GBM-020 | M | 75 | 2010-01-16 | Glioblastoma | O | O | Lt F | 1 | 1 | 0 | GTR | 3 | 1 | 1 | 362 |
| 21 | IRCR-GBM-021 | M | 53 | 2010-05-12 | Glioblastoma | O | O | Rt P | 0 | 2 | 0 | GTR | 2 | 0 | 1 | 173 |
| 22 | IRCR-GBM-022 | M | 64 | 2010-06-28 | Glioblastoma | O | O | Rt F | 0 | 1 | 0 | GTR | 3 | 1 | 1 | 609 |
| 23 | IRCR-GBM-023 | M | 31 | 2010-06-30 | Glioblastoma | O | O | Rt P | 0 | 1 | 0 | GTR | 3 | 1 | 1 | 1147 |
| 24 | IRCR-GBM-024 | M | 56 | 2010-10-13 | Glioblastoma | O | O | Rt T | 0 | 1 | 0 | GTR | 3 | 1 | 1 | 617 |
| 25 | IRCR-GBM-025 | M | 42 | 2010-12-22 | Glioblastoma | O | O | Rt F | 0 | 0 | 0 | PTR | 3 | 1 | 1 | 833 |
| 26 | IRCR-GBM-026 | M | 59 | 2011-03-18 | Glioblastoma | O | O | Lt P | 0 | 0 | 0 | GTR | 3 | 1 | 1 | 672 |
| 27 | IRCR-GBM-027 | M | 31 | 2004-10-08 | Glioblastoma | O |  | Lt F | 0 | 2 | 0 | GTR | 1 | 0 | 1 | 422 |
| 28 | IRCR-GBM-028 | F | 71 | 2011-04-11 | Glioblastoma | O | O | Rt F | 0 | 1 | 0 | GTR | 3 | 0 | 1 | 832 |
| 29 | IRCR-GBM-029 | F | 68 | 2011-06-15 | Glioblastoma | O | O | Rt T | 0 | 1 | 0 | GTR | 3 | 0 | 1 | 465 |
| 30 | IRCR-GBM-030 | F | 70 | 2005-05-09 | Glioblastoma | O |  | Rt F | 0 | 2 | 0 | GTR | 2 | 0 | 1 | 304 |
| 31 | IRCR-GBM-031 | M | 44 | 2012-04-27 | Glioblastoma with oligodendroglioma component | O |  | Lt T | 0 | 1 | 0 | GTR | 1 | 0 | 1 | 407 |
| 32 | IRCR-GBM-032 | M | 74 | 2005-08-22 | Glioblastoma | O |  | Rt F | 0 | 2 | 0 | PTR | 4 | 0 | 1 | 913 |
| 33 | IRCR-GBM-033 | F | 55 | 2006-06-16 | Glioblastoma | O |  | Rt P | 0 | 2 | 0 | GTR | 1 | 1 | 0 | 372 |
| 34 | IRCR-GBM-034 | F | 57 | 2013-07-22 | Glioblastoma | O |  | Lt F | 0 | 0 | 1 | GTR | 1 | 1 | 0 | 367 |
| 35 | IRCR-GBM-035 | F | 65 | 2013-09-21 | Glioblastoma | O |  | Lt F | 0 | 0 | 0 | GTR | 4 | 1 | 1 | 166 |
| 36 | IRCR-GBM-036 | M | 66 | 2007-04-23 | Glioblastoma | O | O | Rt T | 0 | 2 | 0 | GTR | 4 | 0 | 1 | 438 |
| 37 | IRCR-GBM-037 | M | 72 | 2014-04-16 | Glioblastoma | O |  | Rt P | 0 | 0 | 0 | GTR | 3 | 1 | 1 | 536 |
| 38 | IRCR-GBM-038 | F | 58 | 2007-07-04 | Glioblastoma | O | O | Lt F | 0 | 2 | 0 | PTR | 3 | 1 | 1 | 498 |
| 39 | IRCR-GBM-039 | M | 36 | 2007-08-10 | Glioblastoma with oligodendroglioma component | O | O | Rt F | 0 | 2 | 0 | GTR | 3 | 1 | 1 | 1172 |
| 40 | IRCR-GBM-040 | M | 49 | 2007-08-13 | Glioblastoma | O | O | Lt T | 0 | 2 | 0 | GTR | 4 | 1 | 1 | 953 |
| 41 | IRCR-GBM-041 | F | 30 | 2007-08-22 | Glioblastoma | O | O | Rt F | 0 | 2 | 0 | GTR | 4 | 1 | 1 | 740 |
| 42 | IRCR-GBM-042 | M | 57 | 2007-09-07 | Glioblastoma | O | O | Rt F | 0 | 2 | 0 | GTR | 1 | 1 | 1 | 1298 |
| 43 | IRCR-GBM-043 | M | 41 | 2008-03-03 | Glioblastoma | O | O | Rt P | 0 | 2 | 0 | GTR | 3 | 1 | 1 | 352 |
| 44 | IRCR-GBM-044 | M | 68 | 2008-08-20 | Glioblastoma | O | O | Rt P | 0 | 2 | 0 | GTR | 1 | 1 | 1 | 487 |
| 45 | IRCR-GBM-045 | F | 51 | 2008-10-10 | Glioblastoma | O | O | Rt T | 0 | 2 | 0 | GTR | 3 | 1 | 1 | 547 |
| 46 | IRCR-GBM-046 | M | 47 | 2008-10-31 | Glioblastoma | O | O | Lt P | 0 | 2 | 0 | PTR | 4 | 1 | 1 | 523 |
| 47 | IRCR-GBM-047 | M | 65 | 2009-01-07 | Glioblastoma | O | O | Rt F | 0 | 2 | 0 | GTR | 3 | 1 | 1 | 614 |
| 48 | IRCR-GBM-048 | F | 42 | 2009-02-23 | Glioblastoma | O | O | Lt P | 0 | 2 | 0 | GTR | 3 | 1 | 1 | 1747 |
| 49 | IRCR-GBM-049 | M | 51 | 2009-08-22 | Glioblastoma | O |  | Lt F | 0 | 2 | 0 | PTR | 4 | 1 | 1 | 652 |
| 50 | IRCR-GBM-050 | M | 58 | 2009-10-19 | Glioblastoma | O | O | Lt F | 0 | 2 | 0 | GTR | 3 | 1 | 1 | 636 |
| 51 | IRCR-GBM-051 | M | 60 | 2009-10-28 | Glioblastoma | O |  | Rt F | 0 | 2 | 0 | GTR | 1 | 1 | 1 | 985 |

MGMT, [O-6-methylguanine-DNA methyltransferase](https://en.wikipedia.org/wiki/O-6-methylguanine-DNA_methyltransferase), OS, overall survival; Rt, right; Lt, left; F, frontal; O, occipital; T, temporal; P, parietal; CCRT, concomitant chemoradiotherapy; IDH1, Isocitrate dehydrogenase 1; GTR, gross total resection; PTR, partial tumor removal
